# Cathepsin B Levels Correlate with the Severity of Canine Myositis

**DOI:** 10.3390/biom15050743

**Published:** 2025-05-21

**Authors:** Valeria De Pasquale, Emanuela Vaccaro, Federica Rossin, Mariangela Ciampa, Melania Scarcella, Orlando Paciello, Simona Tafuri

**Affiliations:** 1 Department of Veterinary Medicine and Animal Production, University of Naples Federico II, Via F. Delpino 1, 80137 Naples, Italy; emanuela.vaccaro@unina.it (E.V.); fede.rossin@gmail.com (F.R.); orlando.paciello@unina.it (O.P.); simona.tafuri@unina.it (S.T.); 2 Department of Molecular Medicine and Medical Biotechnology, University of Naples Federico II, Via S. Pansini 5, 80131 Naples, Italy; mariangela.ciampa@unina.it (M.C.); melania.scarcella@unina.it (M.S.)

**Keywords:** cathepsin B, myositis, dog, TGFβ-1, SMAD2/3

## Abstract

Cathepsins are protease enzymes vital for normal physiological functions, such as digestion, coagulation, hormone secretion, bone resorption, apoptosis, autophagy, and both innate and adaptive immunity. Their altered expression and/or activity is associated with various pathological conditions, including inflammatory processes. In this study, we investigated the expression levels of cathepsins in muscle specimens collected from dogs affected by inflammatory myopathy (IM) of variable severity established through histopathological analysis. Samples collected from dogs affected by IM at mild, moderate, and severe stages and from healthy (control) dogs were analyzed for the expression profile of 35 proteases using a proteome profiler array. Among the other proteases, cathepsin B was upregulated to an extent depending on disease progression. By exploring the molecular mechanisms underlying the impact of cathepsin B on the disease, we found that the upregulation of cathepsin B in diseased tissues correlates with increased TGFβ-1 expression levels and elevated phosphorylation levels of the TGFβ-1 signaling mediator SMAD2/3. These results suggest that cathepsin B might be involved in the onset and progression of fibrosis commonly occurring in IM diseased dogs. Overall, our findings reveal that modulating cathepsin B activity may hold therapeutic potential for IM.

## 1. Introduction

Cathepsins belong to the family of lysosomal proteases involved in protein degradation and turnover [1,2]. So far, 20 cathepsins have been identified in plants, microorganisms, and animals, including humans. They are classified into serine, cysteine, and aspartate cathepsins based on the key amino acid within their active site, namely, serine (cathepsins A and G), cysteine (cathepsins B, C, H, F, L, K, O, S, V, X, and W), and aspartate (cathepsins D and E) [1]. In addition to their localization in the endo-lysosomal compartment, cathepsins have also been found in the cytoplasm, nucleus, secretory vesicles, and extracellular space, where they cleave and modulate a wide range of biochemically significant signaling pathway intermediates. They are thus capable of regulating several physiological functions, including cell growth, tissue remodeling, coagulation, peptide synthesis, hormone secretion, adipogenesis, immune response, and many other vital processes [2,3,4,5,6]. On the other hand, dysregulation of cathepsin expression and/or activity has been associated with numerous human diseases, including cancer, kidney dysfunction, metabolic and neurodegenerative disorders, and cardiovascular and inflammatory diseases [5,6,7,8,9,10,11,12].

Based on the well-established regulatory activity of cathepsins in various inflammatory conditions [4,5,6,8,13], in this investigation, we explored the potential involvement of these proteases in the onset and progression of canine inflammatory myopathies (IMs) [14]. IM represents a heterogeneous group of muscle diseases characterized by chronic inflammation of muscle tissue, leading to progressive muscle weakness, reduced function, and, in some cases, systemic complications. This group of diseases includes dermatomyositis (DM), polymyositis (PM), autoimmune necrotizing myositis (IMNM), and inclusion body myositis (IBM). Despite clinical and histopathologic differences, one common element is the complex interplay of immune, genetic, and environmental factors leading to the destruction and remodeling of muscle tissue [15].

Despite the progress achieved in recent years, the challenge remains of better understanding the molecular mechanisms underlying IM, which would enable the development of innovative approaches for diagnosing and treating these diseases. Animal models have greatly improved our knowledge of the pathogenetic mechanisms of IM and have proven to be a useful tool for discovering new diagnostic and therapeutic targets [16,17,18,19].

Here, using a proteome profiler array, we evaluated the expression level of distinct proteases in the muscle specimens of dogs affected by IM at varying degrees of severity. Among the tested proteases, we found elevated levels of cathepsin B in the specimens from affected dogs compared to control (healthy) dogs. The expression levels of this cathepsin increased with the progression of the disease, as confirmed by immunohistochemical and Western blotting analyses of clinical specimens. In addition, on the basis of the pivotal role of cathepsins in tissue fibrosis [20], we assessed the expression levels of TGFβ-1 and the phosphorylation levels of the TGF-β signaling mediators SMAD2/3 in the muscle specimens of IM-affected dogs. A close correlation between the upregulation of cathepsin B and increased levels of both fibrosis markers TGFβ-1 and phosphor-SMAD2/3 was found in the advanced stages of the disease.

## 2. Materials and Methods

### 2.1. Selected Cases

Clinical cases of canine IM and normal muscle tissues were selected from the database and tissue bank of the Comparative Neuromuscular Laboratory, Department of Veterinary Medicine and Animal Production, University of Naples Federico II. Chosen cases included IM-affected dog (*n* = 8) and one healthy (control) dog without signs of muscle disease. All diagnoses were based on our routinely performed extensive clinical and laboratory studies [21]. Collected biopsy specimens were frozen in isopentane pre-cooled in liquid nitrogen and stored at −80 °C until further processing for immunohistochemistry. Other biopsy samples from the same animals were directly frozen and stored at −80 °C for biochemical analyses.

### 2.2. Histological and Immunohistochemical Examination

Tissue sections of 8 μm were cut in a transverse plane with a cryostat (−20 °C) and stained according to previously detailed methods [21]. Briefly, hematoxylin and eosin staining was performed to detect histological disorders and inflammatory infiltrates. A previously described inflammation scoring system [22] was used: no inflammation; mild inflammation, 5 to 25 lymphocytes/plasmacytes per high power field (HPF) (400); moderate inflammation, 26 to 50 lymphocytes/plasmacytes per HPF; and severe inflammation, > 50 lymphocytes/plasmacytes per HPF.

Immunohistochemistry (IHC) was performed on muscle samples by applying the horseradish peroxidase (HRP) method using the MACH1 Universal HRP Polymer Detection Kit (Biocare Medical LLC, Concord, CA, USA), as previously described [23]. Sections were stained for mouse anti-cathepsin B (H-5) monoclonal antibody (sc-365558, Santa Cruz, Heidelberg, Germany) diluted at 1:200 in PBS. Normal muscles of a dog of the same size from the archive of the University of Naples Federico II were used as the control.

For each case, ten 20× fields were randomly photographed with Panoramic scan II (3 Dhistech, The Digital Pathology Company, Budapest Öv u. 3 1141, Budapest, Hungary), and each photo was elaborated with Fiji (ImageJ v2.14.0, National Institutes of Health, Bethesda, MD, USA) to quantify cathepsin B expression in each sample.

### 2.3. Proteome Profiler Protease Array

**Scheme 1:**
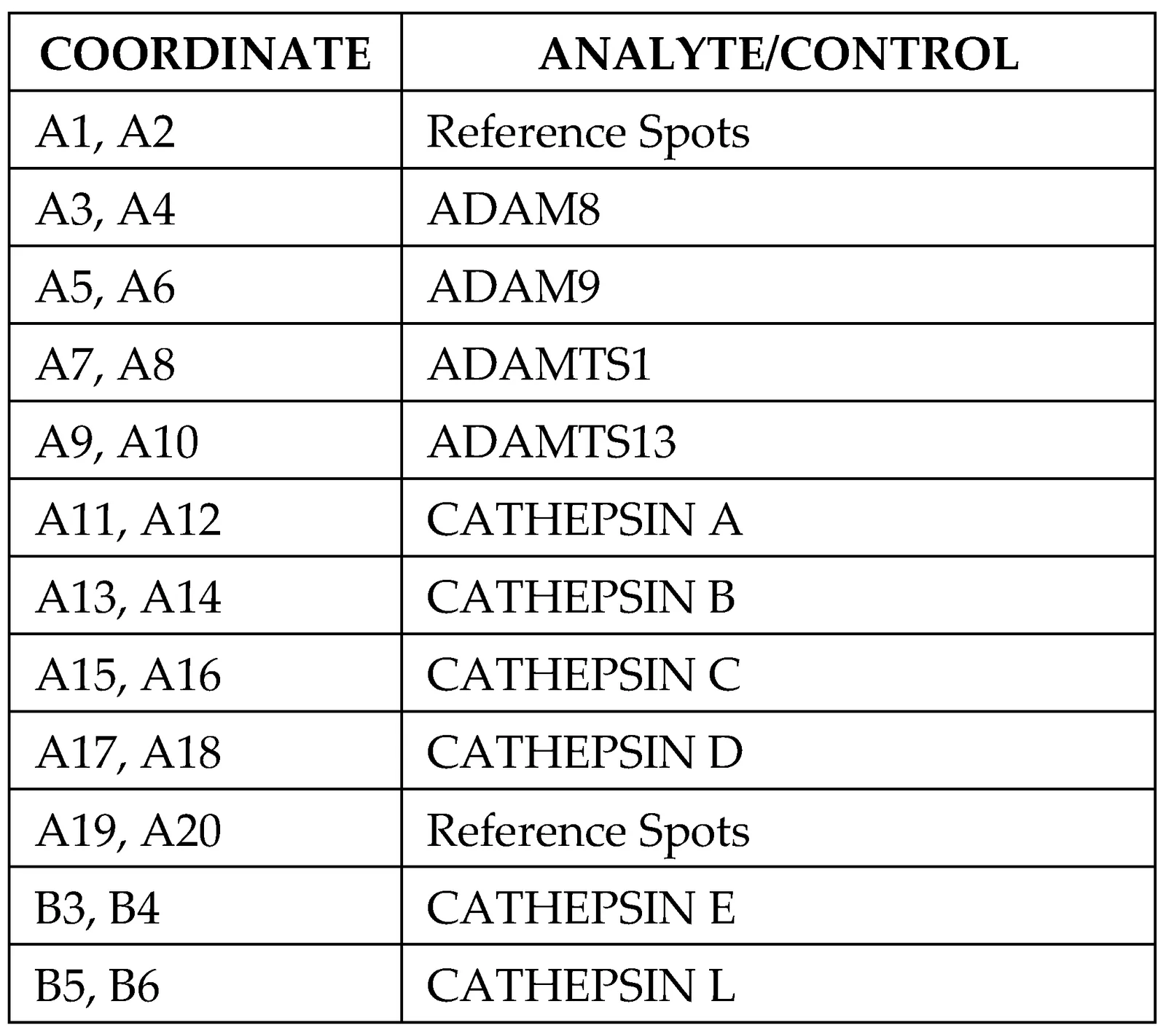

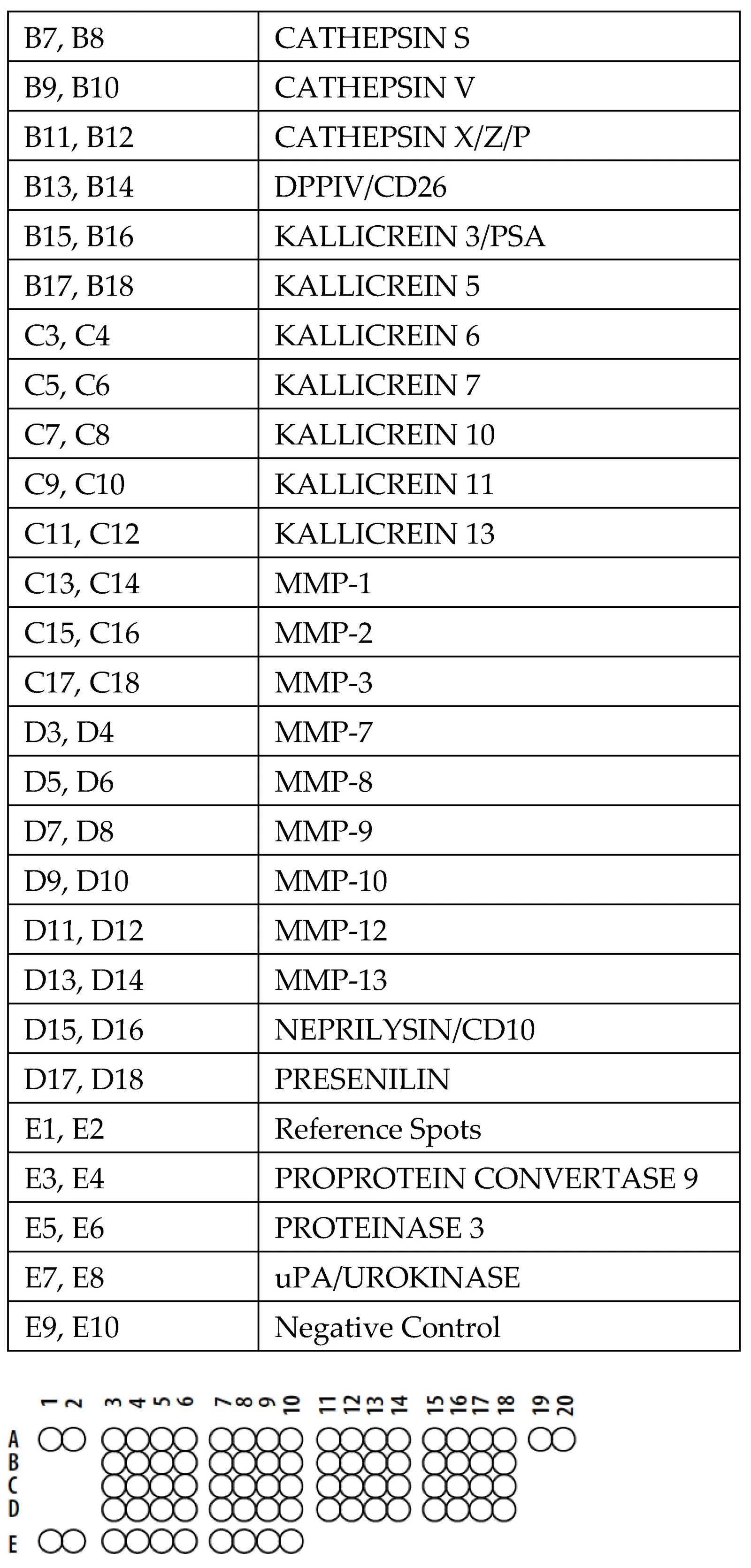
Coordinates of the proteases in the proteome profiler array.

Homogenized muscle tissue samples (100 mg) from IM and control dogs were homogenized at 4 °C in 2 mL of buffer containing 50 mM Tris–HCl pH 7.5, 150 mM NaCl, 1 mM EDTA, 1% Triton X-100, 1 mM EGTA, 10% glycerol, 1 mM β-glycerophosphate, 1 mM phenylmethylsulfonyl fluoride, protease inhibitor cocktail tablet, 1 mM sodium orthovanadate, and 2.5 mM sodium pyrophosphate [16]. Homogenates were centrifuged for 30 min at 14,000× *g* at 4 °C. Supernatants, divided into small aliquots, were stored at −80 °C until use. The total concentration of proteins in each sample was determined using the Bradford assay. A protease antibody array (ARY021B, R&D Systems, Minneapolis, MN, USA) was used to assess the relative levels of selected proteases in the clinical specimens. The list of the proteases and their coordinates is reported in Scheme 1. The array, which allows for screening of different types of proteases, including cathepsins, was performed according to the manufacturer’s instructions, as previously described [24]. Briefly, 200 µg of protein from each sample was incubated overnight at 4 °C with a pre-blocked array membrane. After washing, the array membranes were incubated with secondary antibodies for 30 min at room temperature. Membranes were washed again and exposed with an autoradiography film cassette to an X-ray film for 1–10 min. For data analysis, the positive signal seen on the developed film was identified by placing the transparency overlay template on the array image and aligning it with the pairs of reference spots in the three corners of each array. The array membrane contains two spots for each protease. The spot densities of protease proteins were quantified using ImageJ software. After subtracting background values, the quantified values were normalized to the positive controls on the same membrane. Statistical analysis was performed using the *t*-test on the two values for each sample.

### 2.4. Western Blotting

Muscle homogenates from diseased and healthy (control) dogs, prepared as described in Section 2.3, were subjected to Western blotting analysis. In brief, 50 μg/lane of total proteins was separated on SDS gels and transferred to nitrocellulose membranes. Membranes were treated with a blocking buffer (25 mM Tris, pH 7.4, 200 mM NaCl, 0.5% Triton X-100) containing 5% non-fat powdered milk for 1 h at room temperature. Incubation with the primary antibodies, mouse anti-cathepsin B (H-5) monoclonal antibody (sc-365558), rabbit anti-phospho-SMAD2/3 (pSMAD2/3) polyclonal antibody (sc-11769-R), mouse anti-GAPDH monoclonal antibody (sc-32233 6C5) purchased from Santa Cruz Biotechnologies (Heidelberg, Germany), and mouse anti-TGFβ-1 monoclonal antibody (ab-64715) from Abcam (Cambridge, UK) was carried out overnight at 4 °C. After washing, membranes were incubated with the HRP-conjugated secondary antibodies, goat anti-mouse IgG polyclonal antibody conjugated to horseradish peroxidase (HRP) (sc-2031), and goat anti-rabbit IgG-HRP polyclonal antibody (sc-3837) from Santa Cruz Biotechnology (Heidelberg, Germany) for 1 h at room temperature. Following further washings of the membranes, chemiluminescence was generated using an enhanced chemiluminescence (ECL) kit (Amersham Pharmacia, Buckinghamshire, UK). Densitometric analyses were performed using ImageJ software.

### 2.5. Statistical Analysis

The reported data are expressed as the mean ± SD of at least three experiments. Statistical significance was determined using Student’s *t*-test and the Mann–Whitney U test, a nonparametric test. Student’s *t*-test was used for the analysis of the densitometric array and the blotting profiles of muscle tissues. The Mann–Whitney test was used for statistical evaluation of cathepsin B immunohistochemical positivity in inflammation scoring.

## 3. Results

### 3.1. Histopathological Evaluation of IM Severity in Selected Dogs Compared to a Healthy Sample

Histological examination of muscle biopsies showed significant differences in morphological patterns related to myositis severity. The control case showed no pathology of muscle fibers. Rare lymphocytes and plasma cells were observed in three out of eight muscle biopsies. In three out of eight biopsies, several perivascular and endomysial inflammatory cells were observed.

**Figure 1:**
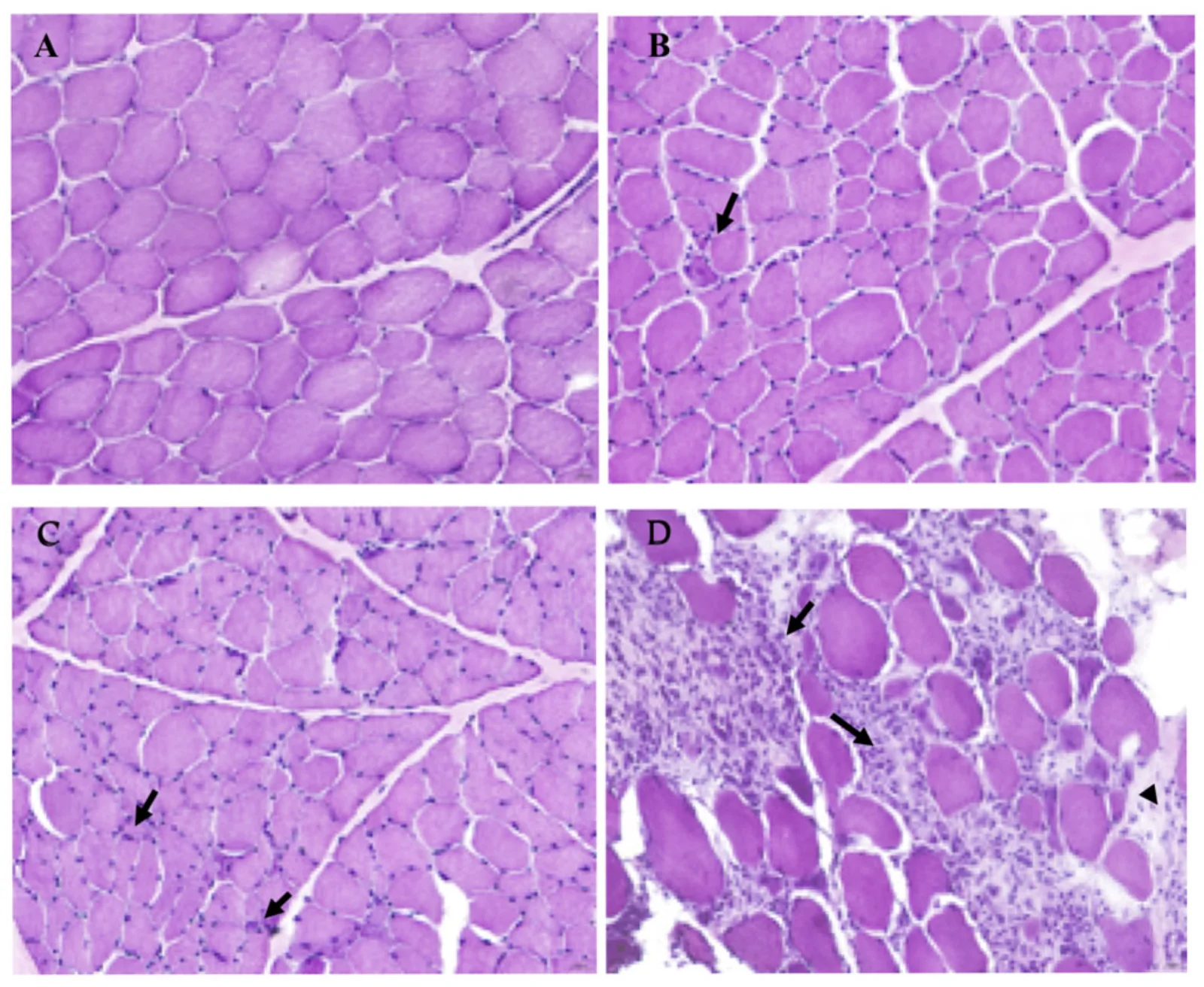
Histological examination of diverse degrees of inflammatory myopathies. (**A**) Control. Absence of inflammatory cells. (**B**) Mild myositis. Rare inflammatory cells in the endomysium. (Arrow). (**C**) Moderate myositis. Single or clustered inflammatory cells in the endomysium (arrows). (**D**) Severe myositis. Numerous groups of inflammatory cells encircled and infiltrated muscle fibers (arrows). Increased endomysial connective tissue (arrowhead). Hematoxylin and eosin staining (original magnification 200×).

In two out of eight biopsies, the endomysium and perimysium were expanded by an inflammatory infiltrate composed mainly of lymphocytes, plasma cells, and macrophages that often encircled muscle fibers. In addition, the endomysium and perimysium were diffusely expanded by fibroblasts associated with an abundant fibrillar extracellular matrix (fibrosis) (Figure 1).

Clinical data and histological diagnoses of all cases are summarized in Table 1.

### 3.2. Proteome Profile Array

**Figure 2:**
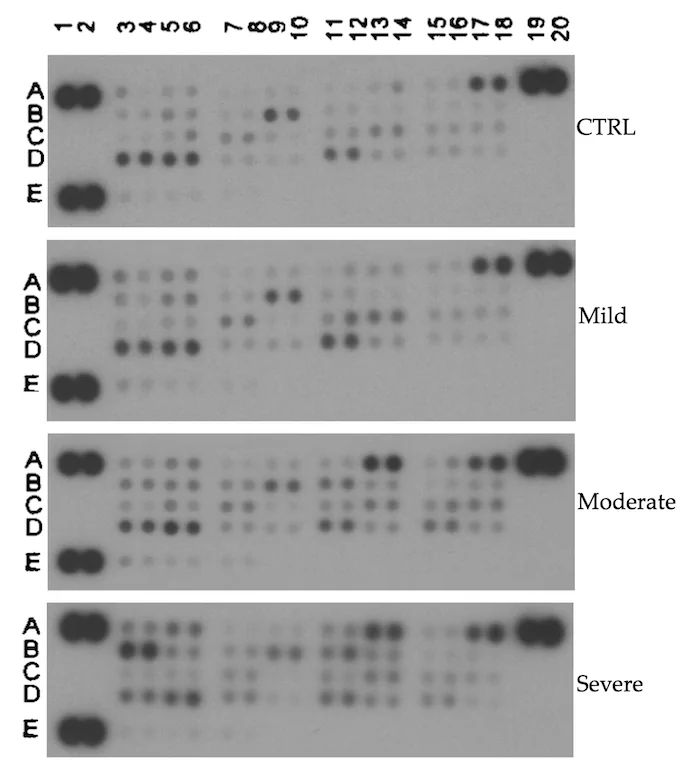
Proteome profiler array—proteases. Representative images of the results from chemiluminescent film images are shown. CTRL, specimens from healthy (control) subjects; mild, moderate, and severe refer to the phenotype of the clinical specimens from muscle tissues of IM-affected dogs. Original Western blot image can be found in Supplementary Materials (Figure S1).

To investigate the expression levels of proteases in muscle specimens from dogs affected by IM at variable degrees of severity compared with healthy (control) dogs, we used a proteome profiler array specific to proteases. Figure 2 reports representative images of the array’s results.

Densitometric analysis obtained using ImageJ software and *t*-test statistical analysis showed an upregulation of various proteases, including distinct cathepsins, such as cathepsins A, B, S, E, and X/Z/P, in the IM muscle samples by comparing spot density between mild vs. healthy (Table 2), moderate vs. healthy (Table 3) and severe vs. healthy (Table 4).

Interestingly, quantification of protease spot densities demonstrated a significant increase in cathepsin B expression levels when comparing grouped samples with moderate/severe IM phenotypes and grouped mild/control (healthy) subjects (Table 5).

The results obtained prompted us to further investigate the impact of cathepsin B on the canine model of IM disease in order to firstly confirm the proteome profiler array data through biochemical analyses of the clinical specimens and next to evaluate whether the upregulation of cathepsin B correlates with fibrosis occurring in the late stages of the disease [16,19,25,26,27].

### 3.3. Upregulation of Cathepsin B Protein Levels in Moderate and Severe IM Muscle Samples

**Figure 3:**
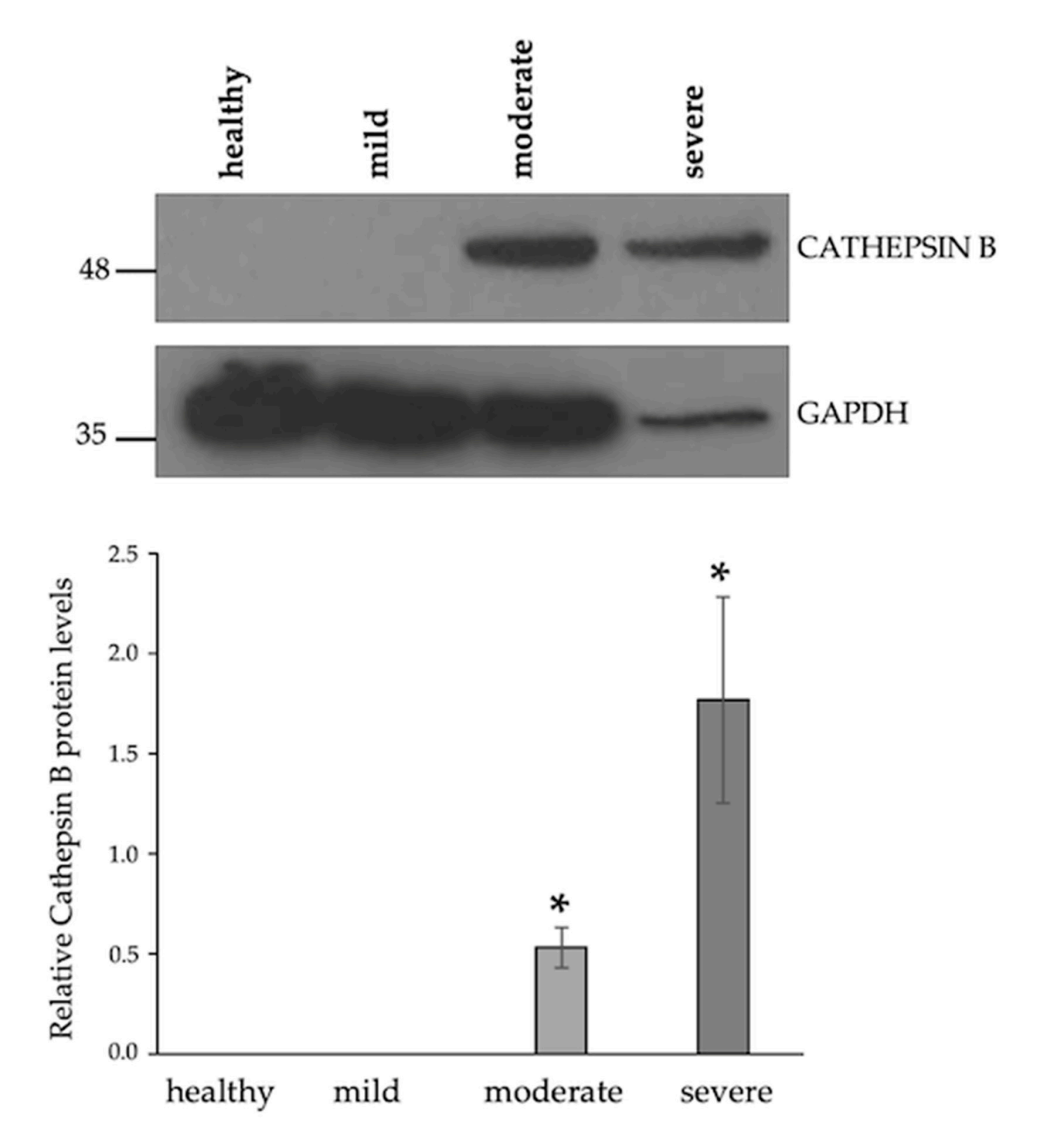
Blotting profile of cathepsin B protein levels in IM clinical specimens. Differences in cathepsin B protein levels are evident in the representative blot. Elevated protein levels of cathepsin B are observed in the samples of moderately and severely affected dogs. The bottom blot shows an equal loading of protein samples in all lanes. Immunoblots are representative of three independent experiments of identical design analyzing one sample from an unaffected dog, three samples from mild IM, three samples from moderate IM, and two samples from severely IM-affected dogs. The histograms report the results of the densitometric analysis of protein bands. * *p* < 0.05. Original Western blot images can be found in Supplementary Materials (Figure S1).

The increasing protein levels of cathepsin B with disease progression, as revealed by the proteome profiler array, was confirmed by Western blotting analysis of the muscle specimens from healthy, mildly, moderately, and severely IM-affected dogs (Figure 3). Densitometric analysis of protein bands showed a significant increase in cathepsin B protein levels in the samples from severely and moderately IM-affected dogs compared to mildly diseased dogs or control (healthy) dogs (Figure 3).

**Figure 4:**
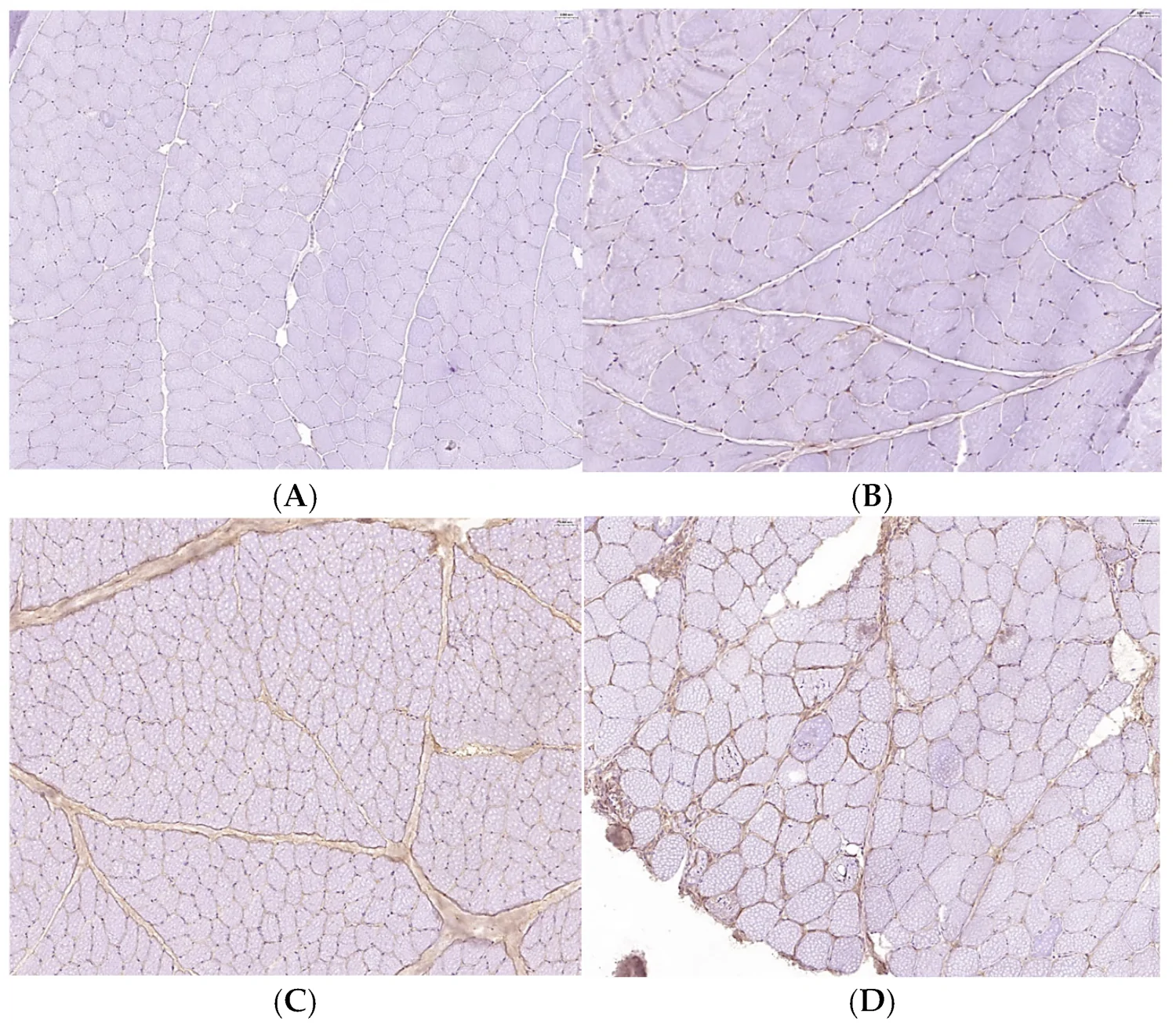
Immunohistochemical positivity for cathepsin B in the control (**A**) and mild (**B**), moderate (**C**), and severe (**D**) myositis. (**A**) The control shows no positivity for the cathepsin B antibody. (**B**) Weak sarcolemmal positivity is observed in mild myositis. (**C**) Muscle tissue with moderate myositis shows sarcolemmal positivity in most muscle fibers. (**D**) In severe myositis, some muscle fibers show intense sarcolemmal, nuclear, and cytoplasmic positivity. (Original magnification 200×).

Immunohistochemistry examination of cathepsin B antibody on tissue sections showed strong sarcolemmal, nuclear, and cytoplasmic positivity in severe cases of IM. In moderate cases, sarcolemmal positivity was observed, and, in mild cases, only weak positivity of muscle fibers was detected (Figure 4).

**Figure 5:**
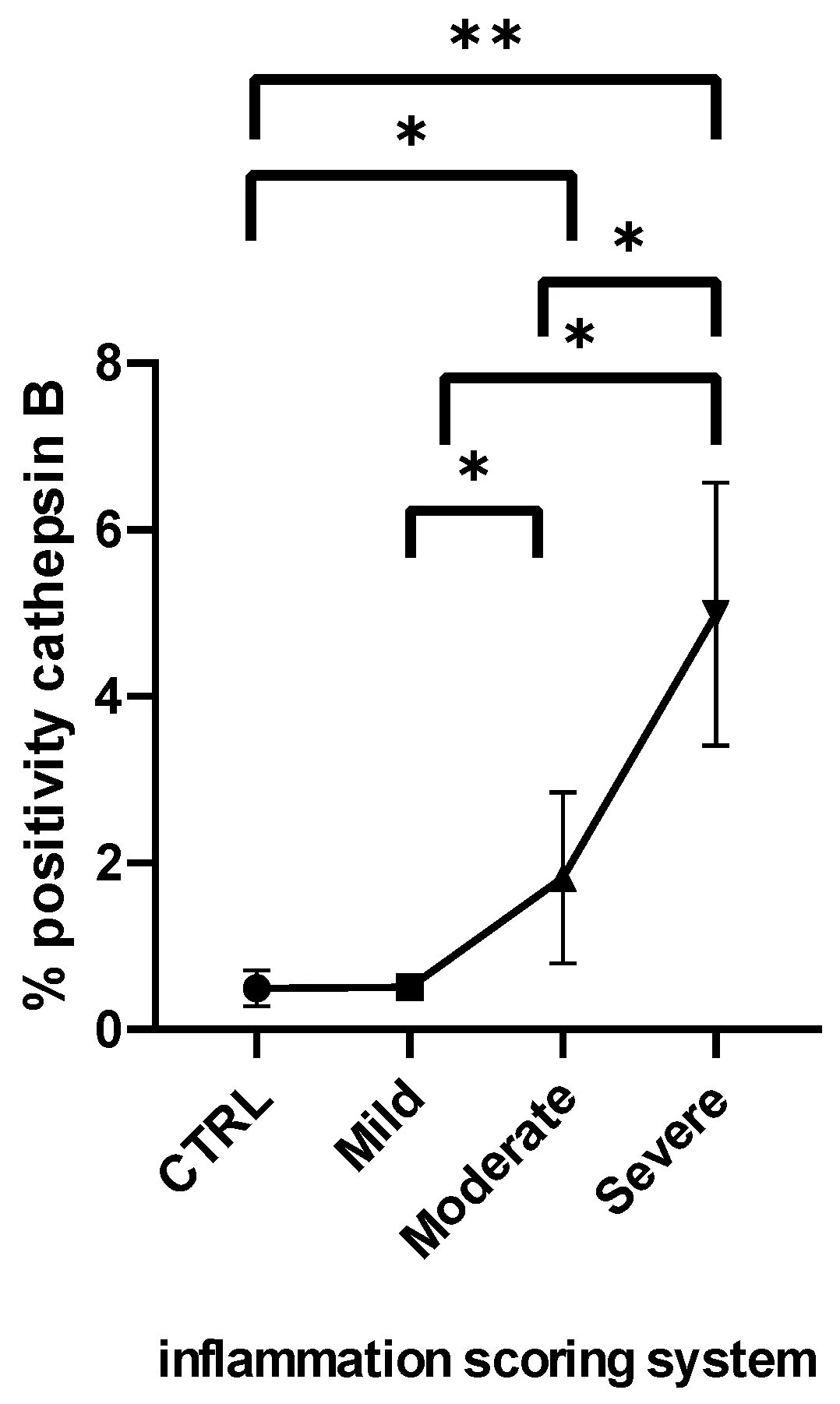
Statistical evaluation of cathepsin B positivity in inflammation scoring. The Mann–Whitney test showed a statistically significant difference among the assessed groups (* *p* < 0.05, ** *p* < 0.01).

Statistical analyses of cathepsin B positivity showed a significant difference between control and severe cases (*p* = 0.0043), between control and moderate cases (*p* = 0.019), between mild and moderate cases (*p* = 0.0286), between mild and severe cases (*p* = 0.0159), and between moderate and severe cases (*p* = 0.0317) (Figure 5).

Overall, we found an enhancement of cathepsin B protein levels as the severity of the disease increased. Indeed, both Western blotting and immunohistochemical analysis of muscle samples from dogs with IM at different stages of the disease showed an increase in cathepsin B expression from mild to severe cases, with statistically significant differences.

### 3.4. Increased Expression Levels of TGFβ-1 and Enhanced Phosphorylation of SMAD2/3 in IM-Affected Dogs

**Figure 6:**
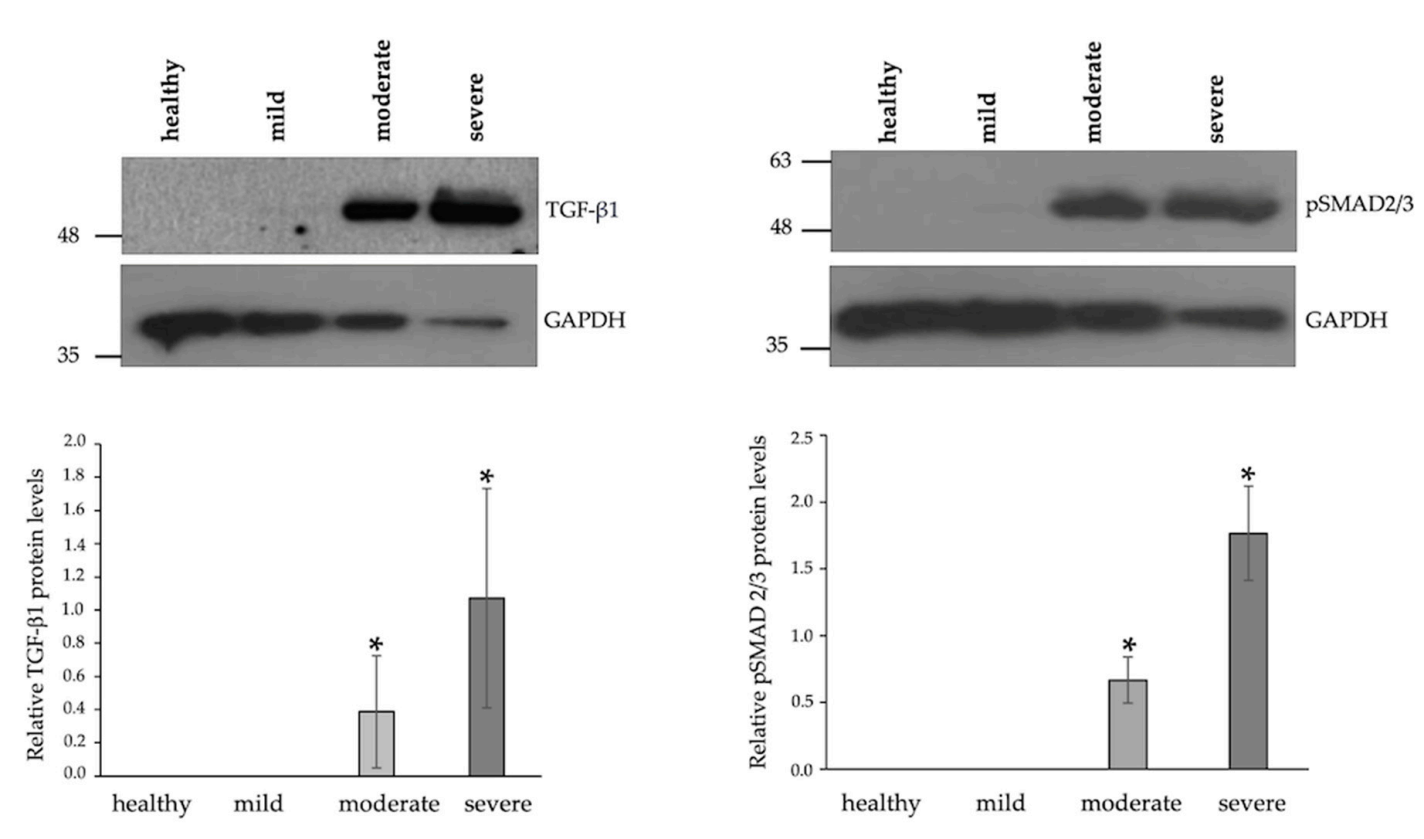
Blotting profile of TGFβ-1 and pSMAD2/3 in canine IM clinical specimens. Differences in TGFβ-1 and pSMAD2/3 levels are evident in the representative blots. Elevated levels of TGFβ-1 and pSMAD2/3 are observed in the samples of moderately and severely affected dogs. The bottom blots show an equal loading of protein samples in all lanes. Immunoblots are representative of three independent experiments of identical design analyzing one sample from unaffected dogs, three samples from mild IM, three samples from moderate IM, and two samples from severely IM-affected dogs. The histograms report the results of the densitometric analysis of protein bands. * *p* < 0.05. Original Western blot images can be found in Supplementary Materials (Figure S2).

Multiple pieces of evidence demonstrate a strict link between cathepsin B activity and TGF signaling in many physio-pathological processes [20,28,29,30]. Thus, we investigated whether the upregulation of cathepsin B would affect TGF expression levels and activation of TGF signaling mediators SMAD2/3. Western blotting analysis of tissues from healthy (control) and affected dogs showed a significant increase in TGFβ-1 protein levels and SMAD2/3 phosphorylation in moderately or severely affected dogs compared to mildly affected or healthy dogs (Figure 6). Interestingly, this effect correlated with the levels of inflammatory infiltrates detected in the samples of IM-tested dogs. Indeed, elevated TGFβ-1 levels are highly correlated with an activated pro-fibrotic pathway, such as TGFβ-1/SMAD signaling, and the progression of many diseases [31].

## 4. Discussion

In this study, we show that cathepsin B protease might be involved in the pathogenesis of IM, thus providing further insights into the molecular mechanisms underlying the onset and progression of the disease. In particular, we demonstrate that cathepsin B is upregulated in the muscle specimens from IM-affected dogs and that cathepsin B protein levels correlate with the degree of severity of IM disease. These data are consistent with previous reports in human IM disease showing upregulation of cathepsin B in specimens from patients with polymyositis and dermatomyositis [32,33]. In addition, using a cellular model of sporadic-inclusion-body myositis, cathepsin B regulation has been shown to be involved in the progression of muscle disease [34]. Interestingly, the administration of the specific cathepsin B inhibitor CA-074Me attenuates apoptosis of myocytes and reduces both inflammation and fibrosis in a guinea pig model of polymyositis [35]. Previous evidence of the important role of cathepsin B in many inflammatory conditions has been reported [8,9,10,11,12,13,28,36,37,38,39,40]. Indeed, in the last decades, an increasing number of pathologies involving inflammatory processes has been associated with impaired synthesis and/or activity of cathepsin B. Cathepsin B, together with cathepsin L, regulates cytokine secretion, expression of NPC2 (Niemann–Pick type C) lysosomal protein, and cholesterol trafficking pathways in macrophages [38]. There is evidence suggesting that lysosomal cathepsin B leakage into the cytosol is critical for NLRP3 inflammasome activation [39]. In the microglia, cathepsin B is associated with the production of interleukin-1β, and this event is considered a major driver of inflammatory brain diseases and brain aging [40]. In addition, cathepsin B has been involved in the progression of diseases like rheumatoid arthritis, liver fibrosis, neurological diseases, inflammatory pain, pancreatitis, hepatitis, myocarditis, cancer, and COVID-19 infection [5,6,7,8,9,10,11,12,13,28,29,30,37,38,39,40,41,42,43,44,45,46].

It is noteworthy that in addition to the increased expression of cathepsin B with disease progression, we also observed the upregulation of other cathepsins, such as cathepsins A, B, S, E, and X/Z/P, in the muscle samples of IM-affected dogs. Although further investigation is needed to better establish the involvement of cathepsins other than cathepsin B in canine myositis, this finding is consistent with several lines of evidence showing a redundancy between different cathepsins in a variety of pathological conditions, including inflammation [47,48,49]. Indeed, cathepsins B and X promote inflammasome-independent, particle-induced cell death during NLRP3-dependent IL-1β activation [47]. Genetic deletion of both cathepsins B and S in a mouse model of autoimmune encephalomyelitis attenuated the clinical phenotype and the progression of the disease, suggesting that inhibition by multiple cathepsins may be required to modulate autoimmune diseases, such as multiple sclerosis [48]. Furthermore, both cathepsins B and S control hepatic NF-κB-dependent inflammation via sirtuin-1 regulation [50]. Interestingly, elevated expression levels of cathepsin S were found in muscle biopsies from IM-affected individuals and in human myoblasts cultured in the presence of IFN-gamma [51]. In this cell type, cathepsin S activity was required for efficient surface display of MHC class II.

In IM, advanced stages are characterized by the replacement of muscle by fibrous tissue [26,27]. In our investigation, we found increased levels of cathepsin B expressed by both muscle fibers, the inflammatory infiltrate, and fibrous tissue, as revealed through histopathological analysis. These findings fit well with our results showing increasing expression levels of TGFβ-1 and phosphorylation of SMAD2/3 as the disease progresses. A correlation between cathepsin B dysregulation and altered TGFβ-1 expression and signaling has been found in many pathological conditions [20,28,29,30,39]. For example, the upregulation of cathepsin B induced by alterations in the TGFβ-1 signaling pathway contributes to the carcinogenic potential of tumor cells [20,28]. On the other hand, cathepsin B and TGFβ-1 are both downregulated in aged skeletal muscle, which is consistent with reduced extracellular matrix remodeling with age [30]. In addition, during extrahepatic cholestasis in mice, liver injury, inflammation, and elevation of indices of hepatic fibrogenesis are cathepsin-B-dependent [44]. The results of our investigation provide the basis for further studies aimed at exploring the molecular mechanisms through which cathepsin B contributes to the pathogenesis of IM.

## 5. Conclusions

Cathepsin B is one of the best-characterized members of the C1 family of papain-like, lysosomal cysteine peptidases, participating in protein degradation and turnover. It displays both exopeptidase, namely, dipeptidyl–carboxypeptidase, and endopeptidase activities under acidic to neutral pH conditions [2,52]. Cathepsin B is ubiquitously expressed in most cell and tissue types. Clear evidence has demonstrated that in addition to its protease activity in the lysosomes, cathepsin B is also localized in other cellular compartments, such as the cytosol, nuclei, and extracellular matrix, where it is involved in a wide variety of pathophysiological processes [3,4,5,6,7]. The establishment of a cathepsin B knockout mouse model contributed to highlighting the fundamental role that the protease plays in both healthy and pathological conditions [53,54]. This has also been made possible thanks to the availability of selective inhibitors of cathepsin B, which include endogenous peptide inhibitors, such as cystatins, thyropins, serpins, and others, and exogenous natural or synthetic low-molecular-weight inhibitors [55,56,57]. Currently, the use of some of these inhibitors, i.e. CA-074, allows us to ameliorate dysfunctional phenotypes in cell and animal models of human disease conditions [5,6,12,42,43,44,55,56,57]. Although further studies are needed, the findings of our investigation on the involvement of cathepsin B in the progression of IM strongly suggest that cathepsin B targeting may have therapeutic relevance for the treatment of IM.

## Figures and Tables

**Table 1:** Clinical data and diagnosis.

Case n.	Sex	Age	Diagnosis
12	M	5 years	Mild chronic lymphoplasmacytic myositis and fibrosis
28	F	6 months	Mild lymphoplasmacytic myositis
31	F	1 year and 2 months	Mild lymphoplasmacytic myositis
21	F	5 years	Moderate and multifocal chronic lymphoplasmacytic myositis
24	M	3 years	Moderate lymphocytic myositis
32	F	5 years	Moderate chronic myositis with fibrosis and severe neurogenic myopathy
7	M	8 years	Severe and diffuse chronic lymphoplasmacytic myositis
27	F	3 years	Severe lymphoplasmacytic myositis
CTRL	M	6 years	No disorders (healthy)

M = male; F = female; CTRL = control.

**Table 2:** Results of densitometric analyses of the proteome array profile of muscle tissues from mildly IM-affected dogs vs. healthy dogs.

Analyte	Fold Change Mild/Healthy	*t*-Test
**CATHEPSIN A**	2.23	0.014
**KALLIKREIN 13**	1.96	0.042
**MMP-10**	1.74	0.006
**KALLIKREIN 6**	1.69	0.006
**PROPROTEIN CONVERTASE 9**	1.70	0.027
**ADAMTS13**	1.65	0.036
**CATHEPSIN X/Z/P**	1.62	0.021
**DPPIV/CD26**	1.61	0.041
**uPA/UROKINASE**	1.58	0.013
**PROTEINASE 3**	1.54	0.015
**MMP-9**	1.50	0.048
**ADAM9**	1.48	0.049

**Table 3:** Results of densitometric analyses of the proteome array profile of muscle tissues from moderately IM-affected dogs vs. healthy dogs.

Analyte	Fold Change Moderate/Healthy	*t*-Test
**CATHEPSIN B**	5.00	0.00001
**CATHEPSIN X/Z/P**	3.41	0.0001
**NEPRILYSIN/CD10**	3.00	0.003
**DPPIV/CD26**	2.82	0.001
**MMP-3**	2.27	0.011
**KALLIKREIN 5**	2.20	0.023
**CATHEPSIN A**	2.16	0.002
**PRESENILIN**	2.13	0.015
**CATHEPSIN S**	2.11	0.005
**CATHEPSIN E**	1.95	0.019
**MMP-9**	1.70	0.016
**KALLIKREIN 6**	1.68	0.011
**ADAMTS1**	1.61	0.049
**MMP-10**	1.60	0.018
**ADAMTS13**	1.58	0.054
**PROPROTEIN CONVERTASE 9**	1.56	0.072
**ADAM9**	1.53	0.035
**uPA/UROKINASE**	1.53	0.009
**PROTEINASE 3**	1.37	0.032

**Table 4:** Results of densitometric analyses of the proteome array profile of muscle tissues from severely IM-affected dogs vs. healthy dogs.

Analyte	Fold Change Severe/Healthy	*t*-Test
**CATHEPSIN E**	5.71	0.00054
**CATHEPSIN B**	4.34	0.00009
**CATHEPSIN X/Z/P**	4.23	0.001
**DPPIV/CD26**	3.85	0.004
**CATHEPSIN A**	3.79	0.004
**MMP-9**	2.60	0.001
**NEPRILYSIN/CD10**	2.32	0.006
**ADAM9**	2.30	0.013
**CATHEPSIN S**	2.18	0.006
**MMP-13**	1.94	0.043
**KALLIKREIN 6**	1.87	0.010
**ADAMTS1**	1.65	0.017
**MMP-10**	1.47	0.055
**uPA/UROKINASE**	1.43	0.047

**Table 5:** Results of densitometric analyses of the proteome array profile of muscle tissues from moderately/severely IM-affected dogs vs. healthy/mild dogs.

Analyte	Fold Change Moderate/Severe vs. Healthy/Mild	*t*-Test
**CATHEPSIN B**	4.38	4.5 × 10^−10^
**CATHEPSIN E**	2.93	0.007
**CATHEPSIN X/Z/P**	2.91	0.000003
**DPPIV/CD26**	2.55	0.0001
**NEPRILYSIN/CD10**	2.46	0.00002
**CATHEPSIN A**	1.84	0.017
**CATHEPSIN S**	1.73	0.0002
**MMP-9**	1.72	0.002
**MMP-3**	1.57	0.012
**ADAM9**	1.54	0.024
**KALLIKREIN 5**	1.54	0.017
**PRESENILIN**	1.53	0.0087
**MMP-2**	1.53	0.017

## Data Availability

The original contributions presented in this study are included in the article/Supplementary Material. Further inquiries can be directed to the corresponding author.

## Supplementary Materials

The following supporting information can be downloaded at: https://www.mdpi.com/article/10.3390/biom15050743/s1, Figure S1: Original Western blot images of Figure 2 and Figure 3; Figure S2: Original Western blot images of Figure 6.

## Abbreviations

The following abbreviations are used in this manuscript:

IMs	Inflammatory myopathies
GADPH	Glyceraldehyde-3-phosphate dehydrogenase
HRP	Horseradish peroxidase
TGFβ-1	Transforming growth factor beta 1
