# Supplementary material for: Cathepsin B Levels Correlate with the Severity of Canine Myositis
Source: Biomolecules. 2025 May 21;15(5):743. doi: 10.3390/biom15050743 (PMC12109109; doi:10.3390/biom15050743)
Supplement: Supplementary file 1 [file biomolecules-15-00743-s001.zip › biomolecules-3508780-supplementary.pdf]

Supplementary Materials

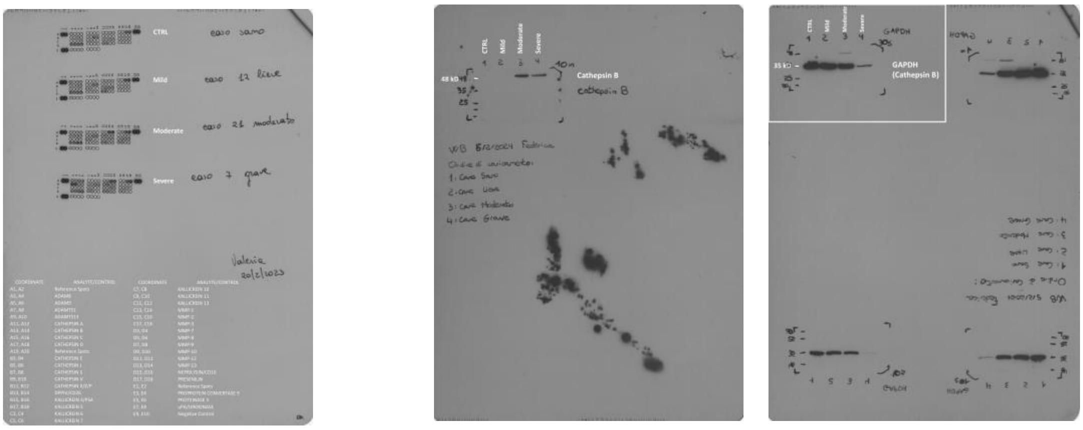

Figure S1. Original Western blot images of Figures 2 and 3.

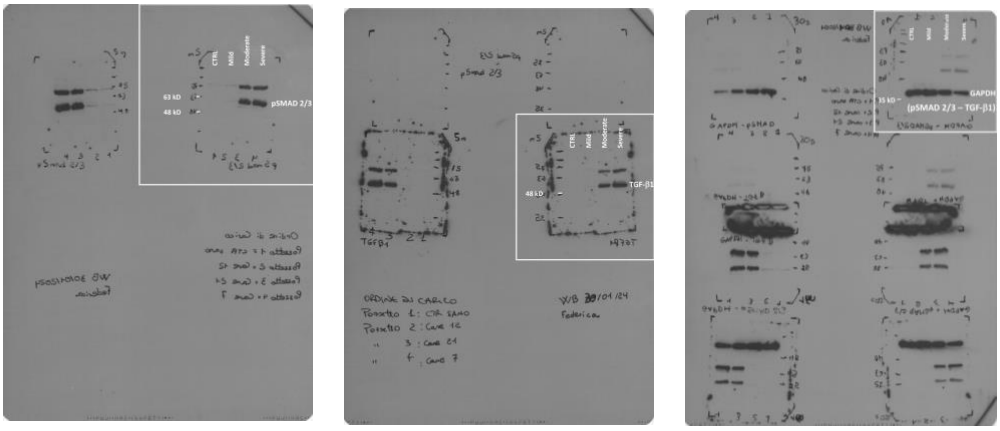

Figure S2. Original Western blot images of Figure 6.
